# Combination of Xuesaitong and Aspirin Based on the Antiplatelet Effect and Gastrointestinal Injury: Study Protocol for a Randomized Controlled Noninferiority Trial

**DOI:** 10.1155/2021/5552506

**Published:** 2021-07-13

**Authors:** Bao-Chen Zhu, Chun-Miao Xue, Rui Lang, Wei-Liang Weng, Xu-Jie Wang, Zhen-Zhen Lei, Sha-Sha Zhang, Wen-Hua Yang, Wan-Tong Zhang, Guo-Dong Hua

**Affiliations:** ^1^Department of Pharmacy, Dongzhimen Hospital, Beijing University of Chinese Medicine, Beijing, China; ^2^Department of Nephrology, Xiyuan Hospital, China Academy of Chinese Medical Sciences, Beijing, China; ^3^Institute of Clinical Pharmacology, Xiyuan Hospital, China Academy of Chinese Medical Sciences, Beijing, China; ^4^National Clinical Research Center for Chinese Medicine Cardiology, Beijing, China

## Abstract

**Background:**

Aspirin is the first-line medication for prevention and treatment of coronary heart disease (CHD). However, long-term use of aspirin resulting in gastrointestinal mucosal injury and bleeding limits the regularity of medication. Xuesaitong is a marketed Chinese medicine contained main active component in Panax notoginseng saponins (PNS), which can significantly inhibit platelet aggregation in patients with CHD. Our previous studies have already showed that PNS could reduce the gastrointestinal mucosal injury caused by aspirin in preclinical study. However, there is a need for further clinical studies to evaluate synergy and attenuation effect of the combination.

**Methods:**

This trial is a prospectively planned, open-labeled, parallel-grouped, single-centered clinical trial. A total of eligible 480 participants will be randomly allocated into three groups: aspirin group, Xuesaitong group, and drug combination group at a ratio of 1 : 1 : 1. The primary outcome is the change of platelet aggregation rate and calprotectin activity. Secondary outcomes include PAC-1, P-selectin, P2Y12, I-FABP activity, and fecal occult blood. *Discussion*. The results of the study are expected to provide evidence of high methodological and reporting quality on the synergy function of Xuesaitong and aspirin upon the antiplatelet and anti-gastrointestinal injury effect for CHD. It also provides an experimental basis for clinical rational drug combination therapy. *Trial Registration*. This trial was registered in the Chinese Clinical Trail Registry, ChiCTR2000036311, on 22 August 2020, http://www.chictr.org.cn/edit.aspx?pid=58798&htm=4.

## 1. Introduction

Coronary heart disease (CHD) is the leading cause of death worldwide, with more than 1.5 million deaths each year [1, 2]. Aspirin is a well-known baseline antiplatelet agent, serving as the first choice for the prevention and treatment of CHD. However, many studies have shown that the long-term administration of aspirin has the potential for significant side effects on the gastrointestinal tract causing mucosal lesions, bleeding, or peptic ulcers [3]. Although enteric-coated aspirin have been developed, 10%–25% long-term users still got peptic ulcers [4] and aspirin increases the risk of gastrointestinal bleeding by almost 3-fold [5]. These side effects limit the regularity of medication which increases the risk of cardiovascular accidents. Therefore, it is particularly important to prevent the occurrence of aspirin-induced gastrointestinal injury. While proton pump inhibitor (PPI) preparations are usually recommended to prevent aspirin-induced gastrointestinal injury [4], especially for the elderly, the application of PPI damages the phospholipid layer in intestinal mucus, which may protect gastric mucosa but increase the risk of small intestinal injury [6]. Therefore, it is urgent to find a strategy to reduce aspirin-induced gastrointestinal injury.

Xuesaitong capsule is a marketed Chinese medicine with components of Panax notoginseng saponins (PNS), which is a mixture of ginsenosides Rb1, Rg1, Rd, Re, and notoginsenoside R1. It has been used in combination with aspirin in preventing cardiovascular disease for several years with a good clinical effect [7, 8]. Our previous study has indicated that the combination could inhibit platelet activation caused by blood hypercoagulability and reduce platelet aggregation to prevent thrombosis [9, 10]. These effects may rely on another finding from our team, which shows that aspirin and Xuesaitong could increase the area under the curve (AUC) and promote the absorption of each other [11, 12]. More interestingly, our research also found Xuesaitong attenuates expression of aspirin-induced tight junction protein and apoptosis of crypt epithelial cells in small intestine [13]. It means Xuesaitong may serve as a pharmacological treatment for aspirin-induced gastrointestinal injury. Since the dual-directional regulation of Xuesaitong, it became the potential combination therapy to solve the clinical dilemma. All the preclinical evidences have elucidated the combination could enhance aspirin's efficacy and decrease aspirin-induced gastrointestinal injury. However, it still lacks high-level clinical evidence.

In this study, we conducted a randomized controlled noninferiority trial to explore whether the efficacy and safety of combination for CHD patients are superior to the single use.

## 2. Methods

### 2.1. Study Design and Setting

This is a prospectively planned, open-labeled, parallel-grouped, single-centered clinical trial. The trial will be conducted at the outpatient care of the Cardiology Department at the Dongzhimen Hospital, Beijing University of Chinese Medicine in China. The staff of the department will be informed of the trial and they will help researchers to recruit participants. The trial is conducted by the ethical principles laid down in the Declaration of Helsinki, the Guideline for Good Clinical Practice, and local laws. The informed consent form will be obtained from all participants before any trial-related procedures were conducted, enrolled after a complete and extensive description of the study. The trial was approved by the Ethics Committee of Dongzhimen Hospital, Beijing University of Chinese Medicine (Supplementary File 1). We present the protocol in accordance with the SPIRIT reporting checklist (Standard Protocol Items Recommendations for Interventional Trials) (Supplementary File 2).

**Figure 1:**
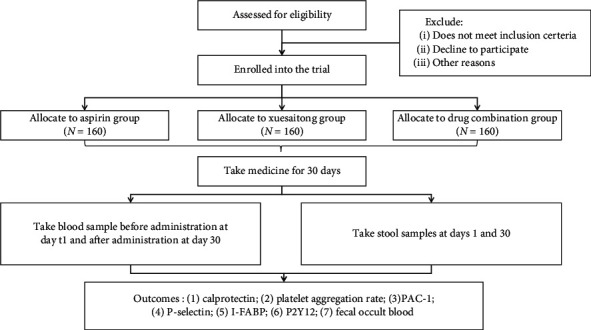
A schematic flowchart of the clinical trial.

In terms of research methods, we refer to and improve one of our previous experimental designs [14]. 480 participants will be randomly assigned to either an aspirin group, a Xuesaitong group, or drug combination group at a ratio of 1: 1: 1. Participants in the aspirin group will be administered aspirin (100 mg, qd) orally 30 min after breakfast, participants in the Xuesaitong group will be administered Xuesaitong soft capsule (contains PNS and excipients; 200 mg, tid) orally 30 min after breakfast, and participants in the drug combination group will be administered both drugs above. The two drugs should be taken half an hour apart, and aspirin should be taken first. All treatment courses will last for 30 days. Blood samples will be acquired 30 min before administration on day 1 and 30 min after administration on day 30. The volume of each blood sample is 4 mL. And fecal samples will be collected on days 1 and 30. Details of patient follow-up are summarized in Table 1 and Figure 1.

### 2.2. Participants

The detailed inclusion, exclusion, and termination criteria are shown in Table 2.

### 2.3. Randomization

This is a single-center clinical trial with a sample size of 480. They were randomly divided into three groups equally. The randomization code will be developed by the statistics office of Dongzhimen Hospital. The trial cases were separated into three arms. The research pharmacy will randomize the participants based on the master randomization list. A third party will join to ensure the allocation concealment.

### 2.4. Sample Size

The sample size was calculated based on the primary outcome (platelet aggregation rate). For power analysis, the primary outcome was assumed to be 38.72% in the aspirin group [15], and 37.79% in the Xuesaitong group [16]. Given a type I error at 0.05 and type II error at 0.2, the required sample size was 131 per arm. Expecting 20% dropout rate, we planned to enroll 160 subjects.

### 2.5. Interventions

The dose and duration of medication in each group are illustrated in Table 3.

1. Aspirin enteric-coated tablets (100 mg, qd) (Bayer S.p.A): a widely used antiplatelet medication
2. Xuesaitong soft capsule (200 mg, tid) (Kunming Huarun Shenghuo Co. Ltd.): the main ingredient of the Xuesaitong soft capsule is Panax notoginseng saponins (PNS), which is commonly used in preventing and treating blood stasis, to prevent cardiovascular disease

### 2.6. Combination

1. Participants are not allowed to take any kind of herbal or chemical medication which have the pharmacological effects of activating blood circulation, preventing platelet aggregation, or anti-inflammatory activity
2. Participants with angina pectoris could take isosorbide mononitrate. In addition, participants could take other medications that have no known drug-drug interaction with aspirin or Xuesaitong soft capsule

### 2.7. Sample Collection

#### 2.7.1. Aspirin Group

Participants should take aspirin with 200 mL of warm water on an empty stomach for 30 consecutive days.

#### 2.7.2. Xuesaitong Group

Participants should take Xuesaitong soft capsule with 200 mL of warm water on an empty stomach for 30 consecutive days.

#### 2.7.3. Drug Combination Group

Participants should take aspirin and Xuesaitong soft capsule on an empty stomach for 30 consecutive days. The interval between taking medicine is 30 minutes.

In all three groups, blood sampling will be carried out before administration on day 1 and after administration on day 30, and fecal samples will be collected on days 1 and 30.

### 2.8. Planned Outcomes and Measurements

The primary outcomes, secondary outcomes, and safety outcomes are summarized in Table 4.

### 2.9. Study Quality Control

Throughout the trial, each participant will be subject to safety monitoring. All the adverse events occurring will be transferred to the office of the clinical trial institution and Ethics Committee, who will review the events and adjudicate them with regards to causality.

We set DSMB (Data and Safety Monitoring Board) for this trial. The member of DSMB comprises physicians, clinical pharmacists, trial methodology experts, statistical experts, and members of the ethics committee, who will conduct risk assessment and safety analysis according to program termination criteria. Also, they will supervise the trial according to a manual of the procedure that has specific information and details for clinical research and laboratory tasks. DSMB are fully blinded to the study intervention reviews and adjudicates adverse events.

### 2.10. Data Management and Statistical Analysis Plan

We use EDC system to collect the data. The system administrator establishes the eCRF and conducts logic verification and range checks for data values. Two investigators fulfill double-data entry.

The analysis will be performed in a modified intention-treat population, which included all randomized participants who received at least one dose of Xuesaitong or aspirin. Continuous variables are presented as the mean ± SD and categorical variables as number and percentage. Comparisons between treatment groups were assessed using Student's *t*-test or Wilcoxon rank-sum test for continuous variables. Comparisons were assessed using the Fisher exact test for categorical variables. Data for patients who were lost to follow-up were censored at the time of the last contact. A multivariable Cox proportional-hazards regression analysis that included center and treatment as independent variables was conducted, and the results are expressed as hazard ratio (HR) with 95% confidence interval (CI). The incidence of adverse events was compared using the chi-squared test. *P* < 0.05 (two-sided) was considered to be statistically significant. All analyses were conducted using SPSS software 26.0.

## 3. Discussion

To our knowledge, this is the first study to investigate the synergy effect of combination of Xuesaitong and aspirin. Aspirin is widely used in clinical practice for primary and secondary prevention of cardiovascular and thrombotic cerebrovascular events [1, 2, 19]. However, the use of aspirin is well recognized to be associated with the risk of some gastrointestinal complications [20, 21]. With the aging of society, the use of aspirin has continued to increase, and the gastrointestinal injury induced by aspirin, even at low doses, has become a clinical problem that needs attention. Some key strategies have been proposed to minimize the aspirin-induced gastrointestinal injury, such as reducing the dose or frequency of aspirin administration, concomitant use of a gastroprotective drug, or even stopping administration of aspirin [22]. But these therapeutic regimens still remain in a dilemma between the prevention of thrombotic diseases and serious gastrointestinal complications. In order to solve this issue, it is urgent to find and establish therapeutic scheme that can not only effectively guarantee the clinical efficacy of aspirin but also prevent gastrointestinal injury. Since the dual-directional regulation of Xuesaitong, it became the potential combination of aspirin to solve the clinical dilemma above.

Xuesaitong, a marketed Chinese medicine with the main active component of PNS, has been widely and long-term used to prevent and treat hematological diseases and cardiovascular diseases [23]. Several clinic trials indicated that Xuesaitong could inhibit platelet activation in patients with blood hyperviscosity syndrome [24]. Interestingly, as for the widespread clinical application together with aspirin, many clinicians found its ability to protect against gastrointestinal injury. In view of this protective efficacy, some researches have shown that Xuesaitong could enhance the proliferative ability of human endothelial cells to heal the wound and stop bleeding [25]. Our previous studies demonstrated Xuesaitong can alleviate gastrointestinal injury caused by aspirin in rats [13]. Combined with HE staining results, our results indicate that the protective mechanism of Xuesaitong on aspirin-induced gastrointestinal injury may act through increasing VEGFA and protecting gastrointestinal mucosa. As for Xuesaitong's ability that both effectively guarantee the clinical efficacy of aspirin and prevent gastrointestinal injury, it is worthwhile to further study the mechanism of the combination of these two drugs and supply a rational combination. Since we have proved that aspirin and Xuesaitong could increase the AUC of each other [11, 12], this means the content of effective drugs in the blood increased. In order to achieve the same blood concentration, the intake of aspirin might be reduced, the adverse reaction might also reduce accordingly. To explore the possibility of reducing the dose of aspirin, it is worth making clear the efficacy and safety of the combination of aspirin and Xuesaitong. By using this randomized controlled noninferiority trial, determining the clinical efficacy indexes like aggregation rate, platelet membrane glycoprotein indexes, and gastrointestinal injury indexes like calcitonin activity and I-FABP allows us to find out whether the platelet aggregation in patients was excessively inhibited and whether the digestive tract injury caused by aspirin is alleviated under drug combination.

In conclusion, the results of this study are expected to provide evidence of high methodological quality on the synergy effect of combination of Xuesaitong and aspirin for CHD synergy effect of combination of Xuesaitong and aspirin.

## Figures and Tables

**Table 1:** Flowchart of a patient follow-up

Group	Screening D-7 to D1	D1	D30	D31 closeout
Patient information	X			
Physical examination	X	X		X
Vital signs	X			X
Blood routine; serum biochemistry; urine routine; stool routine; coagulation routine	X^1^	X^1^		X^1^
ECG	X			X
Stool biomarkers (calprotectin; I-FABP)		X	X	
Blood biomarkers (platelet aggregation; P2Y12; PAC-1; P-selectin)		X	X	

D: day; ECG: electrocardiogram; I-FABP: intestinal fatty acid-binding protein; PAC-1: platelet-associated complement 1. ^1^If the abnormality of this program is clinically significant, if it occurs before screening and at baseline, the subjects will be excluded directly. If it occurs during the observation period of administration, the retest will be followed up until the examination is normal or the abnormality is considered by the study doctor to be of no clinical significance.

**Table 2:** Inclusion, exclusion, and termination criteria.

Criteria
*Inclusion criteria*
(1) 45–75 years of age without gender restriction
(2) Meet the diagnostic criteria of CHD
(3) Meet the diagnostic criteria of grade I–II angina
(4) Meet the diagnostic criteria of blood stasis
(5) Meet the diagnostic criteria of stable angina pectoris
(6) Take regular aspirin (enteric-coated) for at least one year (the incidence of bleeding events, adverse events (AEs), and adverse reactions will be noted)
(7) Calprotectin in stool is above 50* μ*g/g
(8) Before the study start, patients and their families are fully informed and voluntarily willing to sign an informed consent

*Exclusion criteria*
(1) Mental or physical disorders
(2) Pregnant, menstruating, and breastfeeding women
(3) Severe heart disease and severe cardiopulmonary dysfunction
(4) Severe primary diseases, such as cancer, liver and renal damage, and multiple organ failure
(5) Poorly controlled hypertension (>160/100 mmHg)
(6) Current participation in another clinical trial or participation in another clinical trial within three months
(7) Having underwent surgery within eight weeks
(8) Having drug allergy history or allergic constitution
(9) Diabetes
(10) Status epilepticus
(11) Unstable vital signs
(12) Combined with serious infection, infectious diseases
(13) Acute intestinal infection
(14) Unsuitability or poor compliance for this trial

*Termination criteria*
Participant termination
(1) Serious adverse events occur or having obvious abnormal value of the laboratory tests
(2) The investigators believe someone's trial needs to be terminated because of medical, safety, or GCP considerations
Trial termination
(1) Half of the participants have mild adverse events other than gastrointestinal reactions
(2) The investigators believe it is necessary to abort the trial for medical or safety purposes

**Table 3:** Dose and duration of medication.

Group	Medication	Dose and method	Duration
Aspirin group	Enteric-coated aspirin	100 mg, q.d., oral	30 days
Xuesaitong group	Xuesaitong soft capsule	200 mg, t.i.d., oral	30 days
Drug combination group	Xuesaitong soft capsule + enteric-coated aspirin	100 mg q.d. oral aspirin and 200 mg t.i.d. oral Xuesaitong soft capsule	30 days

**Table 4:** Clinical outcomes of the trial.

Items
*Primary outcomes*
Primary outcomes of improving efficacy
Platelet aggregation rate (platelet aggregation analyzer)
Primary outcomes of reducing gastrointestinal side effects
Change in calprotectin activity (ELISA) [17]

*Secondary outcomes*
Secondary outcomes of improving efficacy
(1) Change in PAC-1 activity (flow cytometry)
(2) Change in P-selectin activity (flow cytometry)
(3) Change in P2Y12 acceptor activity (flow cytometry)
Secondary outcomes of reducing gastrointestinal side effects
(1) Change in I-FABP activity (ELISA) [18]
(2) Change in fecal occult blood

*Safety outcomes*
(1) Serum biochemistry
(2) Blood routine
(3) Urine routine
(4) Stool routine
(5) ECG

## Data Availability

The datasets used and analyzed in the current study are available from the corresponding author on reasonable request after the study completion.

## Abbreviations

PNS: Panax notoginseng saponins
CHD: Coronary heart disease
PPI: Proton pump inhibitor
AUC: Area under the curve
I-FABP: Intestinal fatty acid-binding protein
PAC-1: Platelet-associated complement 1
ECG: Electrocardiogram
CCS: Canadian Cardiovascular Society
DSMB: Data and Safety Monitoring Board
HR: Hazard ratio
CI: Confidence interval
VEGFA: Vascular endothelial growth factor A.

## Additional Points

Recruitment started on 2nd of May 2021 (Protocol version 1.0).

## Supplementary Materials

Supplementary Materials 1: approval document of ethics. 2: SPIRIT reporting checklist (Standard Protocol Items Recommendations for Interventional Trials).
