# Supplementary material for: Combination of Xuesaitong and Aspirin Based on the Antiplatelet Effect and Gastrointestinal Injury: Study Protocol for a Randomized Controlled Noninferiority Trial
Source: Evid Based Complement Alternat Med. 2021 Jul 13;2021:5552506. doi: 10.1155/2021/5552506 (PMC8294960; doi:10.1155/2021/5552506)

**Ethics Committee of Dongzhimen Hospital Affiliated to Beijing University of Chinese Medicine**

**Approval Notice Template**

Approval document No.: DZMEC-KY-2019-203

| [project](../../æœ‰é�“/Dict/6.3.69.8341/resultui/frame/javascript:void(0);) [name](../../æœ‰é�“/Dict/6.3.69.8341/resultui/frame/javascript:void(0);) | Research on the mechanism of drug-drug interaction between Panax notoginseng saponins and aspirin based on the esterase enzyme and Intestinal microflora | | | |
| --- | --- | --- | --- | --- |
| Project source | Scientific research project | | | |
| Clinical research unit | Dongzhimen Hospital Affiliated to Beijing University of Chinese Medicine | | | |
| Principal researcher | Zhu Baochen | | | |
| Review category | Primary review | | Review method | Quick review |
| Review date | 2019-12-20 | | Location | Dongzhimen Hospital Affiliated to Beijing University of Chinese Medicine |
| Approval documents | Research program version. No.1.0 Version date 2019-12-16  Informed consent form version. No. v1.0 Version date 2019-12-16  Researcher's resume  GCP certification | | | |
| Review comments | | | | |
| According to “good clinical practice” promulgated by China’s state Food and Drug Administration in 2003, the “Guiding principles of Ethical Review of Drug Clinical Trials promulgated by [National](../../æœ‰é�“/Dict/6.3.69.8341/resultui/frame/javascript:void(0);) [Health](../../æœ‰é�“/Dict/6.3.69.8341/resultui/frame/javascript:void(0);) [and](../../æœ‰é�“/Dict/6.3.69.8341/resultui/frame/javascript:void(0);) [Family](../../æœ‰é�“/Dict/6.3.69.8341/resultui/frame/javascript:void(0);) [Planning](../../æœ‰é�“/Dict/6.3.69.8341/resultui/frame/javascript:void(0);) [Commission](../../æœ‰é�“/Dict/6.3.69.8341/resultui/frame/javascript:void(0);) [of](../../æœ‰é�“/Dict/6.3.69.8341/resultui/frame/javascript:void(0);) [the](../../æœ‰é�“/Dict/6.3.69.8341/resultui/frame/javascript:void(0);) [People](../../æœ‰é�“/Dict/6.3.69.8341/resultui/frame/javascript:void(0);) ‘ [s](../../æœ‰é�“/Dict/6.3.69.8341/resultui/frame/javascript:void(0);) [Republic](../../æœ‰é�“/Dict/6.3.69.8341/resultui/frame/javascript:void(0);) [of](../../æœ‰é�“/Dict/6.3.69.8341/resultui/frame/javascript:void(0);) [China](../../æœ‰é�“/Dict/6.3.69.8341/resultui/frame/javascript:void(0);) in 2016, and the ethical review of “declaration of Helsinki”and CIOMS, upon review of the Ethics committee, the review result is “approve”, and the specific comments are as follows:  Please follow the principle of GCP and the approval research program to conduct the clinical research.protect the safety and rights of the subjects.  Please complete the clinical trial registration before the start of the research.  If there is any modification of clinical trial protocol and informed consent or replacement of the principal researcher, you need a re-review and execute after obtaining an approval  If there is any serous adverse event, you shall promptly report to the Ethics committee.  According to the regulations of the ethics committee's annual/periodic review frequency, the applicant should submit progress reports 1 month prior. The sponsor units shall be reported to the leader of the ethics committee to submit the summary report of each center research progress. When any may significantly affect the trials, or increase the risk of the subjects, the applicant in a timely manner, please submit a written report to the ethics committee.  If the research includes subject that does not meet the inclusion criteria or conform to the exclusion criteria or conform to the requirement of suspension test, drops out of the study, givie wrong therapy or dose, give solutions such as follow on the prohibited drug combination research, Or may  impact  on the subjects' rights and interests with violation of GCP principles, the sponsor / researchers should submit protocol deviation report.  please timely submit suspension/termination of the study when the applicant plan to suspend or terminate clinical research,  please finish the report and submit research, summary the findings and conclusions of the report when you complete clinical research, | | | | |
| annual/periodic review frequency | | Please submit progress reports 1 month prior 2020-12-22 | | |
| Contact number | | Shang Jianwei 010-84012709 | | |
| Ethics Committee | | Dongzhimen Hospital Affiliated to Beijing University of Chinese Medicine | | |
| Date | | 2019-12-20 | | |


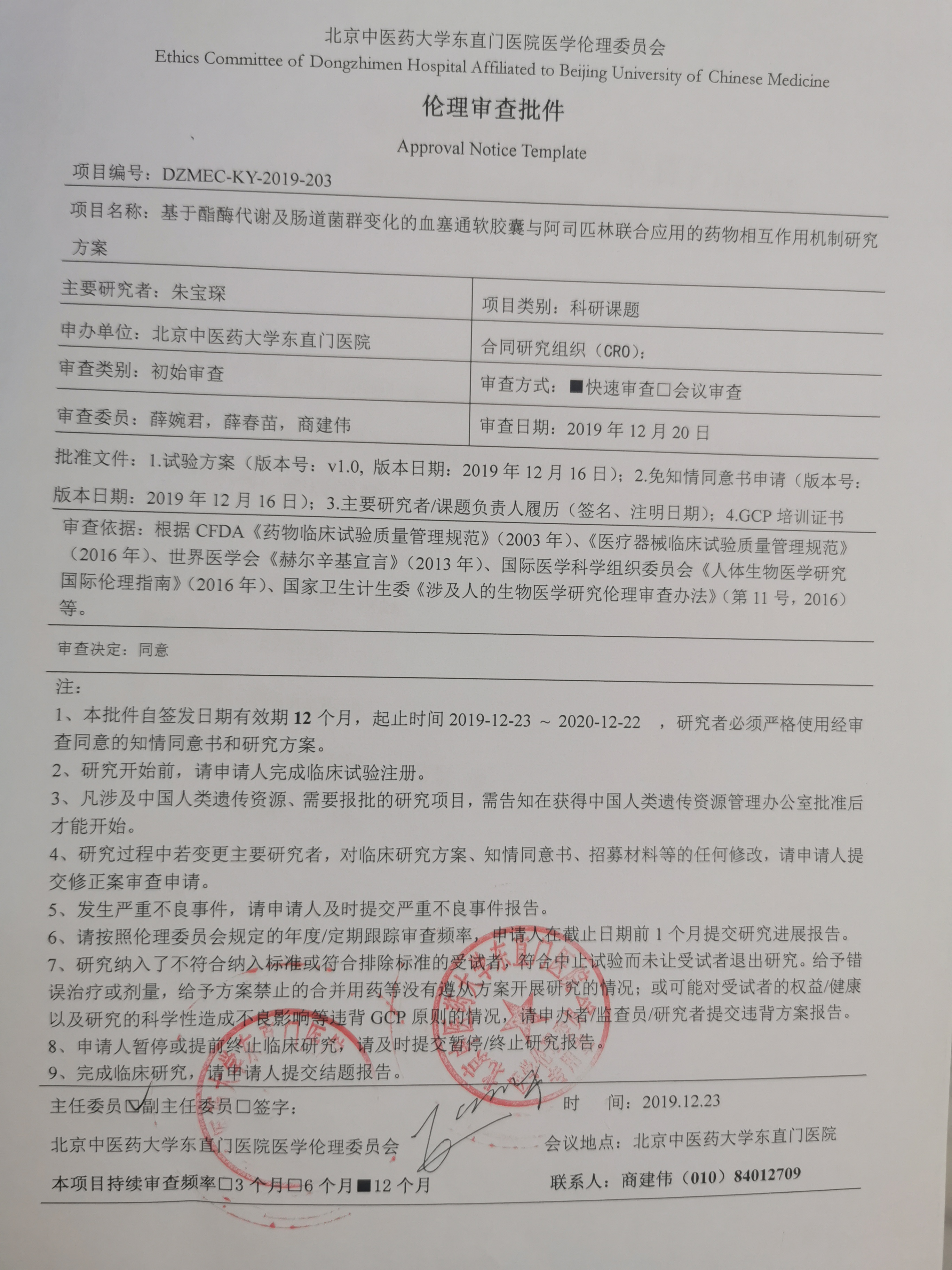

Supplement: Supplementary Materials — 1: approval document of ethics. 2: SPIRIT reporting checklist (Standard Protocol Items Recommendations for Interventional Trials). [file 5552506.f1.zip › 5552506.f1/Supplementary file 1 approval document of ethics.docx]
